# Toxic Effects of Zinc Chloride on the Bone Development in *Danio rerio* (Hamilton, 1822)

**DOI:** 10.3389/fphys.2016.00153

**Published:** 2016-04-29

**Authors:** Antonio Salvaggio, Fabio Marino, Marco Albano, Roberta Pecoraro, Giuseppina Camiolo, Daniele Tibullo, Vincenzo Bramanti, Bianca M. Lombardo, Salvatore Saccone, Veronica Mazzei, Maria V. Brundo

**Affiliations:** ^1^Experimental Zoo-prophylactic Institute of SicilyCatania, Italy; ^2^Department of Veterinary Science, University of MessinaMessina, Italy; ^3^Department of Biological, Geological and Environmental Science, University of CataniaCatania, Italy; ^4^Department of Biomedical and Biotechnological Sciences, University of CataniaCatania, Italy

**Keywords:** zebrafish, zinc chloride, bone development, skeletal malformations, calcein

## Abstract

The increase of heavy metals in the environment involves a high exposure of aquatic organisms to these pollutants. The present study is planned to investigate the effects of zinc chloride (ZnCl_2_) on the bone embryonic development of *Danio rerio* and confirm the use of zebrafish as a model organism to study the teratogenic potential of this pollutant. Zebrafish embryos were exposed to different ZnCl_2_ concentrations and analyzed by ICP-MS. The skeletal anomalies were evaluated to confocal microscope after staining with calcein solution and RhodZin^TM^-3,AM. The data show a delay in hatching compared with the controls, malformations in the process of calcification and significant defects in growth. In conclusion, the current work demonstrates for the first time the Zn toxic effects on calcification process and confirm zebrafish (*Danio rerio*) as suitable alternative vertebrate model to study the causes and the mechanisms of the skeletal malformations.

## Introduction

In recent years, there has been an increasing ecological and global public health concern associated with environmental contamination by heavy metals. Aquatic organisms are exposed to a significant amount of pollutants, especially to heavy metals derived from geogenic, industrial, agricultural, pharmaceutical, and domestic effluents (Heath, 1987) that lead to biochemical disturbances (Dethloff et al., 1999; Orun and Talas, 2008; De Domenico et al., 2011; Copat et al., 2012; Guerriero et al., 2014; Fasulo et al., 2015).

Environmental contamination can also occur through metal corrosion, atmospheric deposition, soil erosion of metal ions, and leaching of heavy metals, sediment re-suspension, and metal evaporation from water resources to soil and ground water (Skidmore, 1965). Natural phenomena such as weathering and volcanic eruptions have also been reported to significantly contribute to heavy metal pollution (Fergusson, 1990; Goyer, 2001; Bradl, 2002).

Since heavy metals are required for various biological process but are also toxic at high levels, they represent an interesting object of research.

The Zinc (Zn) is an essential element for organisms and plays an important role in aquatic physiological processes (Watanabe et al., 1997). However, excessive Zn in the aquatic environments is toxic (Huang et al., 2010; Zheng et al., 2011). The fish toxicity by Zn has been well documented in various fish species (Dautremepuits et al., 2004; Giardina et al., 2009).

Relatively little attention has been paid to zinc's role in human nutrition and health (Skidmore, 1965). The Zinc may play a role in various biological processes, such as in enzyme activities, cell structures, protein structures, and carbohydrate metabolism in the fishes. Physiological and biochemical alterations were reported in the early life stage (ELS) of fishes after exposure to Zn, such as chorion structure and permeability changes and inhibition of enzyme activities in organs (Küçükoğlu et al., 2013). Zinc toxicity changes during the course of embryonic development of fish; in fact, embryonic toxicity in the absence of chorion was greater than in its presence. In addition, zinc toxicity to the ELS of fishes can be easily influenced by water properties such as temperature, dissolved oxygen concentration, hardness, pH, salinity, osmoregulation, and water permeability (Guner, 2010; Zhu et al., 2012).

This study was planned to investigate the effect of ZnCl_2_ on the bone embryonic development of zebrafish and to determine if it could represent a model for investigation ofteratogenic potential of environmental pollutants.

*Danio rerio* is a model vertebrate extensively used in scientific investigation worldwide (Zhu et al., 2012; Alsop and Wood, 2013; Howe et al., 2013; Yin et al., 2014). The US Environmental Protection Agency in fact designated zebrafish as a powerful vertebrate model for assessing environmental contaminants and itwas selected to evaluate the toxicity during development. In the last decades, protocols and techniques have been developed in order to evaluate the effects of chemicals at different levels of biological organization of this species and to evaluate the lethal and sub-lethal effects of pollutants.

## Materials and methods

### Zebrafish embryos

All experiments have been carried out at Centre for Experimental Fish Pathology of Sicily (CISS), University of Messina, Establishment for Users recognized by the Italian Ministry of Health, according to the Italian Law D.L. 2014, n° 26, in application of the 2010/63/UE. This project had been registered with the serial number CISS/17/2013 and had been authorized according to the former National Law D.L. 116/92.

The study was conducted in wild-type zebrafish embryos kept, reproduced and contaminated within a plant “Zebtec Tecniplast Stand Alone” at the CISS. After the pairing of one male with two females and the spawning, contaminated and control embryos were evaluated as described by Westerfield (1995) and Kimmel et al. (1995).

### Exposure procedures

Three Hundred embryos, in three repetitions, were exposed to different ZnCl_2_ concentrations for 21 days starting from zygote stage. In order to determine the range of ZnCl_2_, threshold tests were performed and we used the following ZnCl_2_ concentrations: 200, 150, 100, 50, 25, 10, 5, 2.5, 1, and 0.5 mg/L. The control samples (30 larvae in three repetitions) were kept in water, in the same conditions. The larvae were analyzed with Leica M205C microscope.

### Calcein solutions

Calcein solution (0.2%) was prepared by dissolving 2 g of calcein powder (Sigma, Life Sciences) in 1 liter of deionized water. Due to calcein's strong acidifying effects, an appropriate amount of Sodium hydroxide (NaOH; 0.5 N) was added to the solution to have the pH 7.4. Treated zebrafish embryos were netted and immersed in the solutions in Petri dishes for 10 min. After the immersions, the embryos were rinsed in fresh water for 10 min in order to eliminate the excess of calcein. The embryos were then euthanized in 3% solution of tricaine-methanesulfonate (MS222) and mounted on glass slides. Vectashield mounting medium (Vector Laboratories, Inc., Burlingame, CA, USA). Observations were carried out using confocal laser scanning microcopy (CLSM; Zeiss LSM 700), equipped with the ZEN-2011 software.

### Staining with Rhodzin^TM^-3,AM

Some larvae, after having been treated with calcein, were anesthetized and fixed in a solution of 3.7% formaldehyde (pH 7.0), for 45 min. After washing in PBS, the samples were treated with Triton 1X, incubated for 20 min in a blocking solution and after incubated for 1 h with RhodZin^TM^-3,AM (INVITROGEN, 1:4) in the dark. After repeated washing, the samples were mounted on glass slides. Vectashield mounting medium (Vector Laboratories, Inc., Burlingame, CA, USA). The observations were made using confocal laser scanning microcopy (CLSM; Zeiss LSM 700), equipped with the ZEN-2011 software.

### Zinc analysis

Zinc concentrations in larvae were determined byInductively Coupled Plasma Mass Spectrometry (ICP-MS). Samples were digested in 65% Nitric acid (HNO_3_; Carlo Erba Chemicals) overnight. After digestion, the samples were diluted by the addition of ultra-pure water (Merck) and analyzed by ICP-MS and compared with standards.

### Statistical analysis

Statistical analysis was made with Prism Software (Graphpad Software Inc., La Jolla, CA, USA). Data were expressed as mean or *SD*. Statistical analysis was carried out by two-way ANOVA test. A *p*-value of 0.05 was considered to indicate a statistically significant difference between experimental and control groups.

## Results

The exposure to ZnCl_2_ induced a delay in hatching compared with the controls in a concentration-dependent manner. At concentrations >200, the 100% of the eggs not haching from the chorion. At the 200 mg/L of ZnCl_2_ the hatching from the chorion was failed in 90% of fertilized eggs.

**Figure 1:**
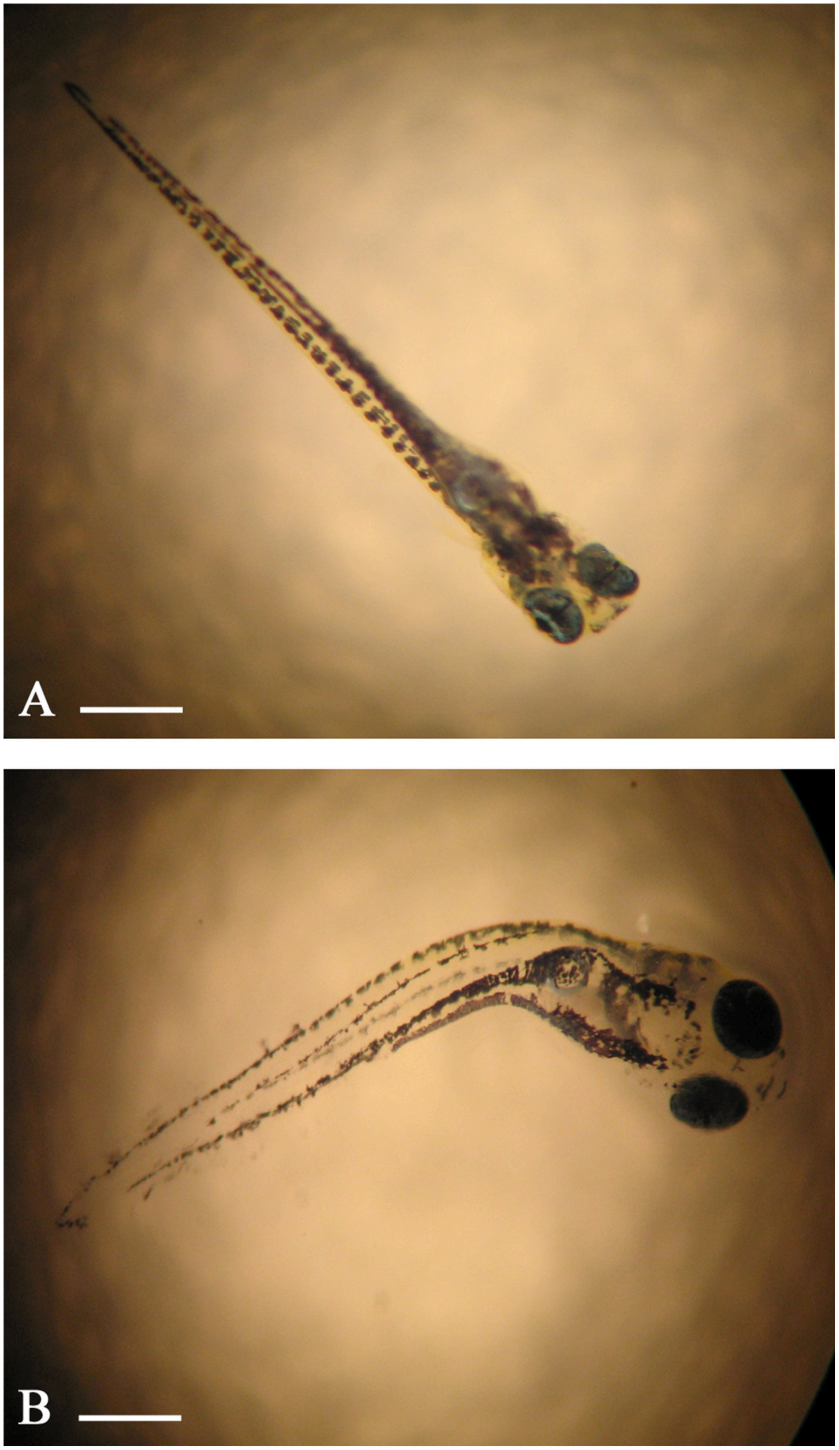
**Embryo zebrafish observed with the stereomicroscope**. **(A)** Larvae treated with 10 mg/L of ZnCl_2_; curvatures of the spine are not evident. **(B)** Larva treated with 200 mg/L of ZnCl_2_ with pronounced curvature of the spine. Scale bar: **(A)** = 100 μm; **(B)** = 120 μm.

After contamination with increasing concentrations of ZnCl_2_ skeletal malformations can be observed without histochemical assay because of the transparency of embryos *D. rerio*. These anomalies were not observed at lower concentrations (0.5–25 mg/L; Figure 1A), whereas the treatment with the higher concentrations (50–200 mg/L) of ZnCl_2_ induced abnormalities of the spine with lateral curvature similar to a scoliosis (Figure 1B). Moreover, the fishes treated with ZnCl_2_ showed a different size in a concentration-dependent manner highlighting defects in growth.

The skeletal anomalies were evaluated to confocal microscope after staining with calcein solution and RhodZin^TM^-3,AM. No fluorescent signals could be detected in embryos up to 4 days post-fertilization (dpf). First fluorescent signals became apparent in 5-dpf embryos and were restricted to the head. The calcein staining of axial skeleton in the trunk region first appeared on day 7-dpf.

**Figure 2:**
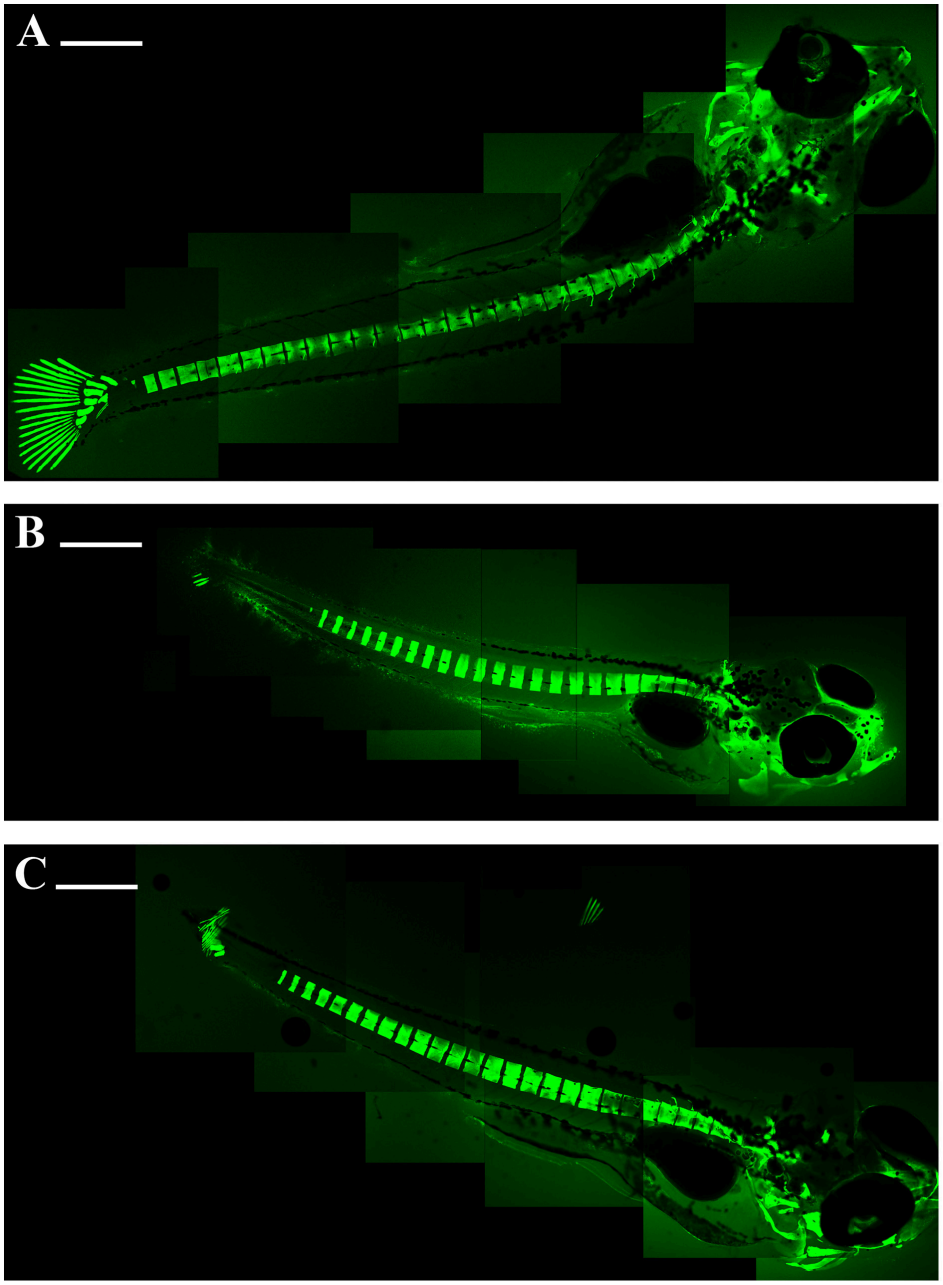
**Embryo zebrafish 15 days treated with calcein solution**. The decalcification of the vertebrae is evident; the gill arches and caudal fins show more damage with increasing concentration. Composite images of the embryos were assembled with Adobe Photoshop 5.0. **(A)** 50 mg/L; **(B)** 100 mg/L; **(C)** 200 mg/L. Scale bar: 500 μm.

The fusion of the hemal spinal with caudal axis was identified in embryos contaminated with Zn. Skeletal abnormalities, like kyphosis, were observed in fifth cranial vertebrae, in first prehemal arch and in fifth–sixth hemal arches (Figures 2A–C).

**Figure 3:**
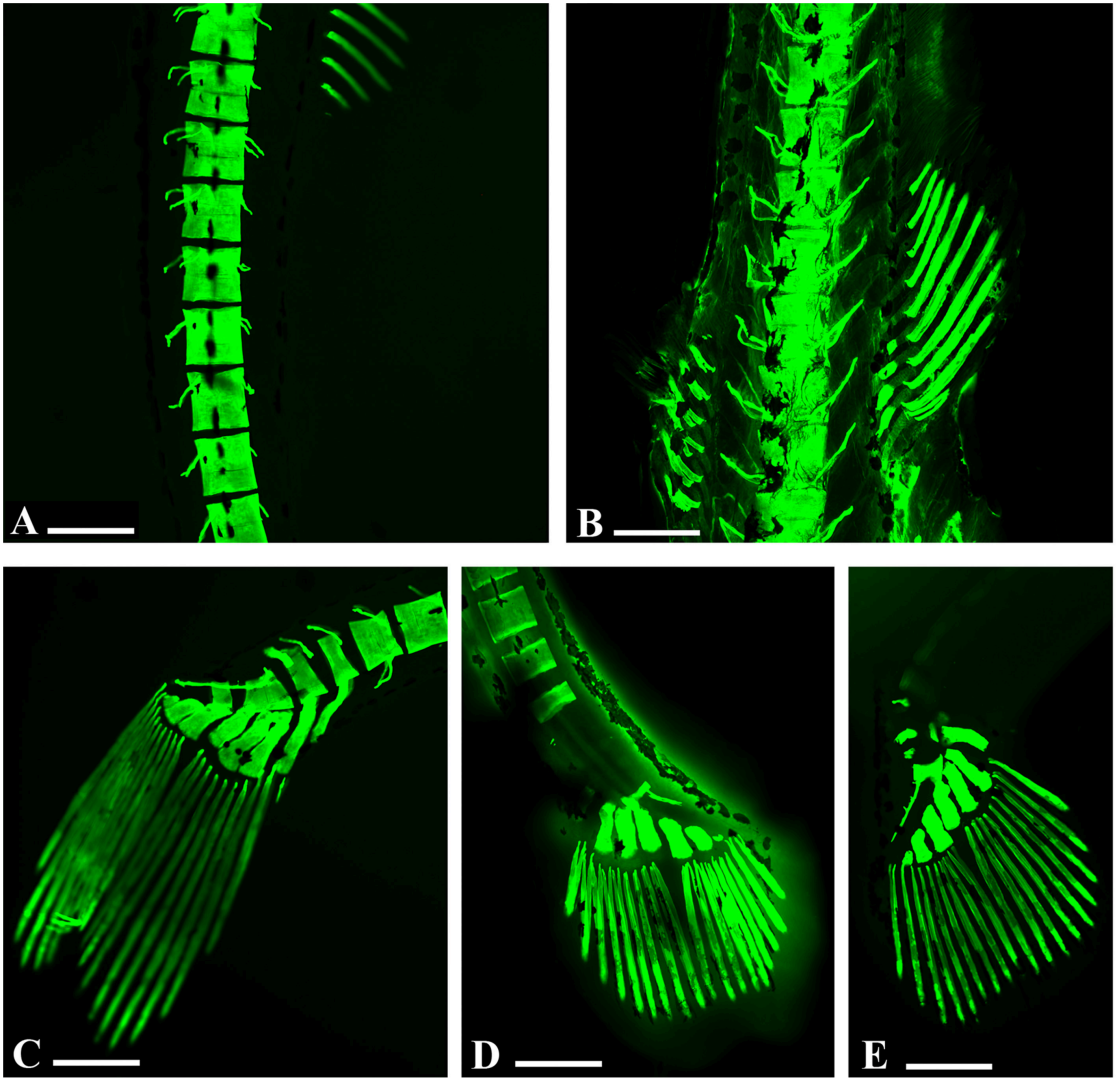
**Embryo zebrafish treated with calcein solution. (A,B)** Particularly abnormalities in vertebral spines in samples treated with 200 mg/L. **(A)** Zebrafish embryos 16 days. **(B)** Zebrafish embryos 21 dpf. **(C–E)** Zebrafish embryos 16 days. Details of the abnormalities of the caudal rays with clear areas of decalcification of caudal vertebrae related to the concentrations tested. **(C)** 50 mg/L; **(D)** 100 mg/L; **(E)** 200 mg/L. Scale bar: 200 μm.

**Figure 4:**
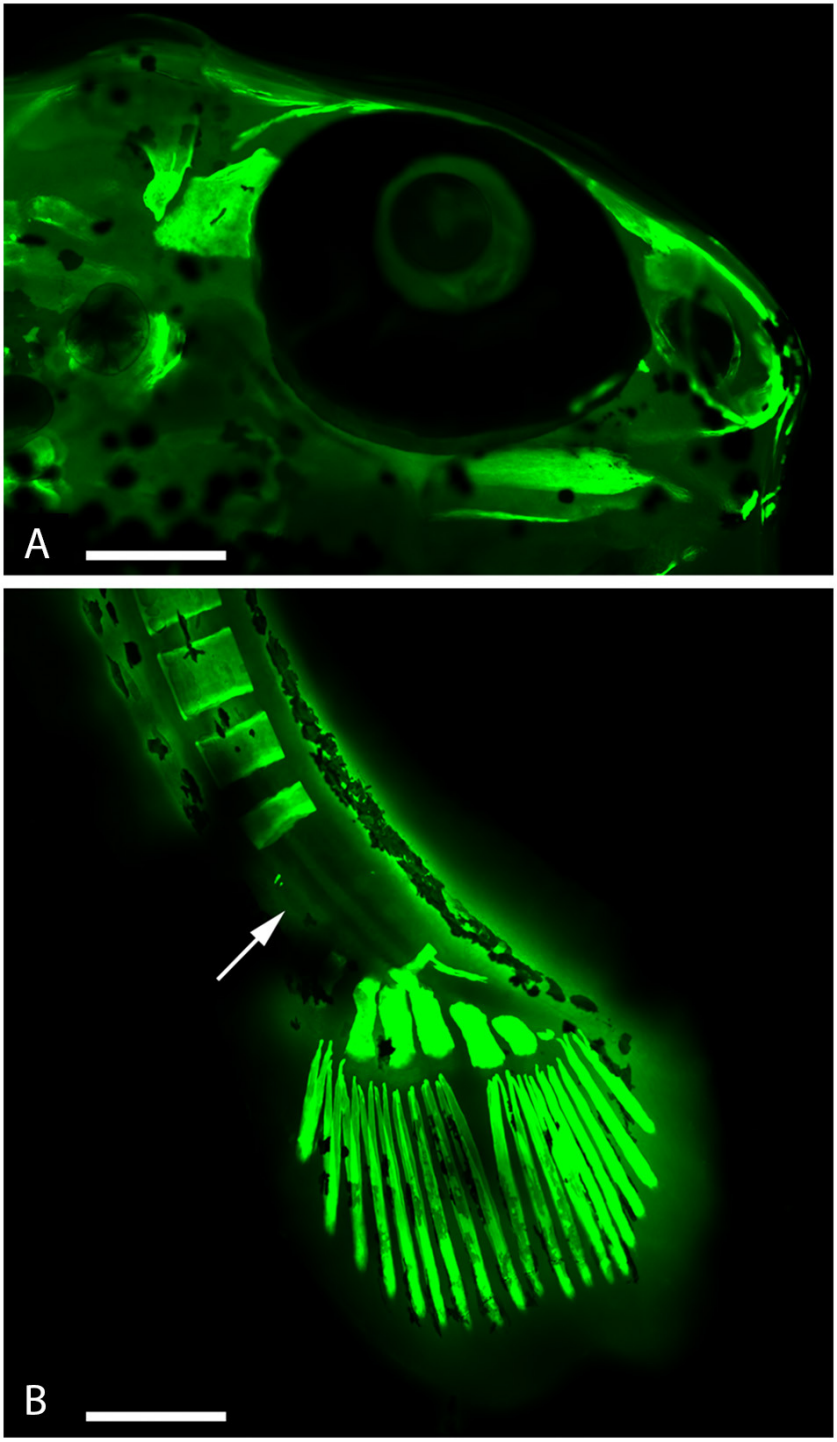
**Embryo zebrafish treated with calcein solution and RhodZin^TM^-3,AM. (A)** It is evident a considerable tapering of the muzzle. **(B)** Presence of zinc in the areas of decalcification (arrow). Scale bar: 200 μm.

We observed alterations in the vertebral spine (Figures 3A,B), in the caudal fin (Figures 3C–E) and in some bones of the skull such as in the premaxillary and dental bones, with a considerable tapering snout (Figure 4A).

Incubation with RhodZin^TM^-3,AM, indicated that the alterations affecting the skeletal system seems to be linked to a decreased calcification because of the replacing of calcium with zinc (Figure 4B).

**Figure 5:**
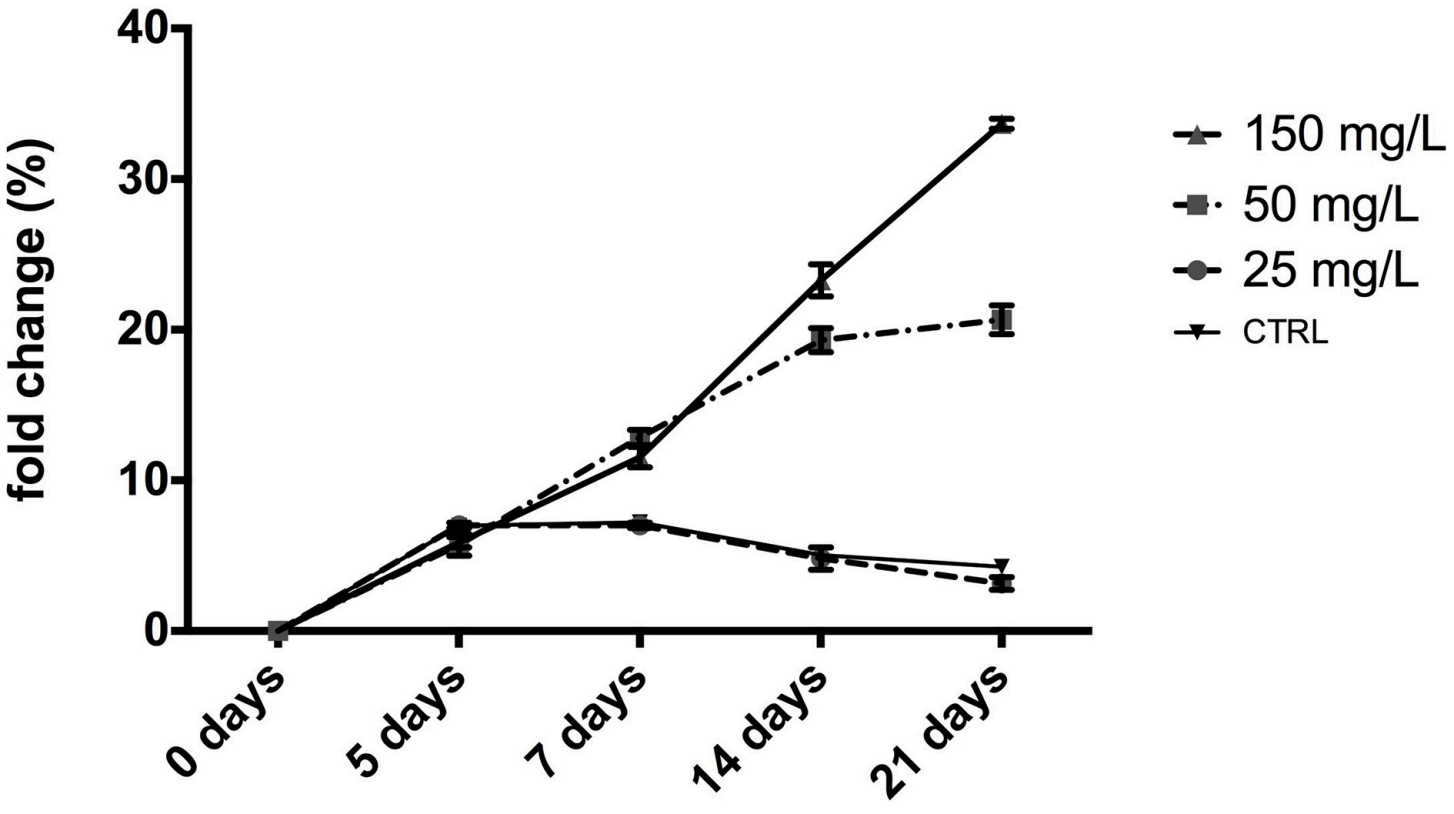
**Fold change of zinc concentrations in zebrafish embryos (mean ± *SD*)**. The samples contaminated with low concentrations of ZnCl_2_ 25 mg/L, do not present differences with the control samples (untreated); athigher concentrations (50 and 150 mg/L of ZnCl_2_) the Zn levels was increased after 7 days of contamination (*p* < 0.0001) respect to control (*p* < 0.0001). Values are expressed as a mean ± *SD* of four independent experiments performed in triplicate. Statistically significant differences by two-way analysis of variance (ANOVA) were performed.

The ICP-MS analysis was used to evaluate the Zn levels. We observed that 25 mg/L ZnCl_2_ did not modify the Zn levels in larvae respect to control (untreated group; Figure 5). At higher concentrations (50 and 150 mg/L) after 7 days we observed a significant increase of the levels of Zn respect to untreated samples (CTRL) in a dose dependent manner (*p* < 0.0001; Figure 5).

## Discussion

Although the toxicity of zinc to several fish species has been documented, the toxicity of this metal is not well-known for all aquatic organisms (Küçükoğlu et al., 2013). Nevertheless, a number of studies have indicated that zinc toxicity is species-specific and varies with developmental stages (Ho et al., 2012). In this study we showed cytoskeleton an modifications induced by Zn exposure in Zebrafish, demonstrating also that *D. rerio* is a good animal model for these investigations.

The inhibition of DNA synthesis is the most likely explanation of Zn teratogenicity but the specific mechanism is unknown (Jakovac et al., 2015). Some authors affirmed that in mammals an excessive amount of zinc chloride is little teratogenic to due to protective mechanisms of the maternal liver and metalloenzymes (Küçükoğlu et al., 2013), while for a oviparous aquatic organisms, not having no protection, an excessive amount of zinc chloride may cause an abnormal development (Nagel, 2002). Our data showed that environmental factors may induce skeletal malformations (Du et al., 2001) as well as shown from high rate of morphological alterations identified in our vivo model.

The calcification process in zebrafish was found to progress from the anterior to posterior regions in a segmented fashion as the embryos developed. It was interesting to note that the anterior-to-posterior calcification process of vertebrae was not continuous, but instead appeared to be divided into two distinct domains: an anterior domain and a posterior domain. The rationale for dividing them into two domains is based on observations that vertebrae numbers 2 and 3 were always calcified later in time than vertebrae 4 (Du et al., 2001). Calcification process was interrupted in some vertebral areas after Zn exposure. Furthermore, abnormalities were detected also in the bone of the skull, in particular, in premaxillary and dental bones with a considerable tapering snout, because of what the eyes of some larvae looked more large.

It has been demonstrated that zinc induces defects in the hemiarches, dorsal-base, and ventral-base leading to torsion and scoliosis of the spine (Du et al., 2001). We observed that substitution of calcium with zinc in the bone induced a curvature of the spine (scoliosis) more evident during the various stages of development.

In conclusion, the current work demonstrates for the first time the Zn toxic effects on calcification process and confirms zebrafish (*D. rerio*) as ideal alternative vertebrate model to study the causes and the mechanisms of the skeletal malformations.

## Author contributions

AS, FM, and MVB developed the research idea and experimental design. MA managed fish facility. RP and GC conducted experiments. BL made the statistical analysis. SS made the pictures to the confocal. AS, VM, MVB, DT, and VB co-wrote the manuscript and all authors read and approved the final manuscript.

### Conflict of interest statement

The authors declare that the research was conducted in the absence of any commercial or financial relationships that could be construed as a potential conflict of interest. The reviewer BR and handling Editor declared their shared affiliation, and the handling Editor states that the process nevertheless met the standards of a fair and objective review.
